# Gas flux cyclic regime at an open vent magmatic column inferred from seismic and acoustic records

**DOI:** 10.1038/s41598-019-42033-z

**Published:** 2019-04-05

**Authors:** Gen Kondo, Hiroshi Aoyama, Takeshi Nishimura, Maurizio Ripepe, Giorgio Lacanna, Riccardo Genco, Ryohei Kawaguchi, Taishi Yamada, Takahiro Miwa, Eisuke Fujita

**Affiliations:** 1 Graduate School of Science, Hokkaido University, Sapporo, 060-0810 Japan; 2 Faculty of Science, Hokkaido University, Sapporo, 060-0810 Japan; 3 Graduate School of Science, Tohoku University, Sendai, 980-8578 Japan; 4 Dipartimento di Scienze della Terra, Università di Firenze, Firenze, 50121 Italy; 5 Meteorological Research Institute, Japan Meteorological Agency, Tsukuba, 305-0052 Japan; 6 National Research Institute for Earth Science and Disaster Resilience, Tsukuba, 305-0006 Japan

## Abstract

On August 7, 2014, a new effusive vent opened on the northern flank of Stromboli. A characteristic pattern was observed in both seismic and infrasonic signal amplitudes prior to this effusive eruption. The pattern consisted of the repeating cycle: (1) quiet phase, (2) puffing phase, and (3) explosion phase. Correlation between seismic and infrasound signal suggests that pulses in the puffing phase were caused by repetitive bursts of small gas pockets at the central crater, while the explosion phase coincided with an explosion at the central crater. We show that degassing of the magma column occurred in cycles of increasing gas flux, which controlled the transition from a bubbly flow (puffing phase), to a slug flow (explosion phase) gas regime. The quiet phase was characterized by a constant time length of 150 s, indicating that the gas rose in the magma column as well-organized waves of gas layers. These cycles represent cyclic changes of the gas flux regime in the shallow magma column, associated with increases in the magma-gas supply input rate before the effusive eruption.

## Introduction

**Figure 1:**
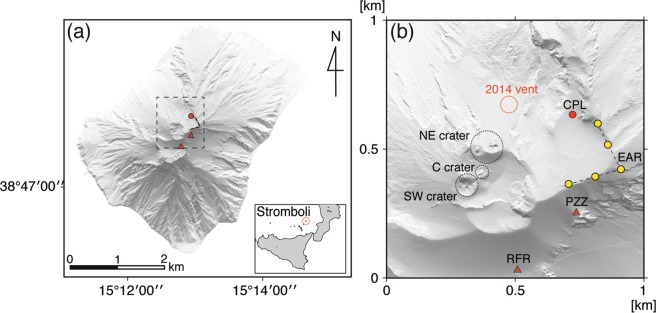
(**a**) Map of Stromboli volcano, showing location of the seismic stations (red triangles), tiltmeter (red circle) and infrasonic array (yellow circles) used in this study. (**b**) Close-up of the crater area. Black dashed circles indicate main craters (SW: southwest crater, C: central crater, NE: northeast crater), and red open circle indicates the position of new effusive vent that opened on August 7, 2014. The map of Stromboli was created using Generic Mapping Tools ver. 4.5.15 (ref.^34^).

Stromboli Volcano, located in the South Tyrrhenian Sea, Italy, is well known for the repetitive emission of volcanic gases with basaltic magma from its summit vents (Fig. 1a). The term *Strombolian activity* refers to various types of surface activity, from relatively mild degassing to vigorous ejection of volcanic bombs, lapilli, and ash^1^. Many observational, theoretical, and experimental studies have been conducted to understand the complex interaction between magma and gas in the conduit, which is at the origin of the Strombolian activity^1–6^.

Explosive activities at Stromboli are classified into three types according to frequency and intensity: normal explosions (herein referred to as “explosions”), major explosions, and paroxysms^7^. In addition to these activities, moderate and discrete gas emissions are observed in active vents, a process referred to as puffing^6,8^. These crater explosive activities are sometimes (1985, 2002–2003, 2007 and 2014) interrupted by lava effusions in the northwest Sciara del Fuoco (SDF) slope of Stromboli’s edifice^9^.

On August 6, 2014, lava overflowed from a fissure on the outer slope of the summit crater after a few months of increased surface activity^10^. On the next day, at 5:15 UTC (hereafter, time is given in UTC), a new flank vent opened at 650 m above sea level on the SDF, and lava effusion started^11^ (Fig. 1b). This effusive activity continued until November 13, 2014, with the total volume of lava ejected estimated to be 7.4 × 10^6^ m^3^ (refs^10,12^).

Although several studies have focused on the long-term seismic activity preceding and during the lava effusions in the past^13,14^, the short-term seismic activity has not been studied in detail. About two months before the 2014 lava effusion activity, we installed temporary seismic and tilt stations around the summit crater. Our instruments successfully recorded the beginning and the end of volcanic activity related to the transition from the explosion at the summit to the effusive lava flow on the flank. Detailed analysis of seismic and infrasonic data shows a distinctive cycle of waveforms from July 27 to August 6, one day before the onset of the lava effusion on the flank. As the distinctive cycle emerged in the period just before the transition of volcanic activity, it may reflect a temporal change in the physical conditions of the shallow conduit system. Therefore, in this study, we examine the signal’s characteristics and its distinctive cycle, before then investigating its long-term activity. Finally, we present a schematic model based on the features of our geophysical data and previous studies on Strombolian activity.

## Results

From May 19, 2014 to June 6, 2015, we deployed one broadband seismometer and one tiltmeter at two temporary stations (RFR and CPL) located within 500 m of the summit craters (Fig. 1b). The seismometer installed at RFR was a Trillium Compact (Nanometrics Inc.), which has a natural period of 120 seconds. The tiltmeter installed at CPL was a T701-2A biaxial bubble type sensor (Applied Geomechanics Inc.). Analog signals from these sensors were digitized by HKS-9550 data loggers (Keisokugiken Corp.) at a sampling frequency of 100 Hz with 24 bit resolution. Data were stored in a compact flash memory card with time marks synchronized to GPS signals. In addition to these sensors, we use seismic data from a broadband CMG-40T seismometer (Güralp Systems) at station PZZ, and the 5-element microphone array at EAR. These instruments are part of a permanent monitoring network run by the University of Florence (Fig. 1b). Data from the PZZ seismometer and EAR infrasonic array were digitally recorded by the data logger of Güralp Systems at a 100 Hz sampling frequency, with GPS synchronized time marks.

During the normal activity of Stromboli, explosions occurred at the three active vents^10^, northeast (NE), central (C), and southwest (SW), as shown in Fig. 1b. In late May 2014, when we installed our instruments, the eruptive activities in the SW crater were characterized by gas-rich emissions with some short lasting (<10 s) incandescent bombs. In contrast, the eruptions from the NE crater were ash-dominated explosions with longer durations. Activity at the C crater was as usual characterized by intense puffing^15,16^ with small magma fragments being ejected a few meters above the vent. During this period, the averaged explosion rate was 16–17 explosions per hour^10^.

### Seismic and acoustic cyclic patterns

**Figure 2:**
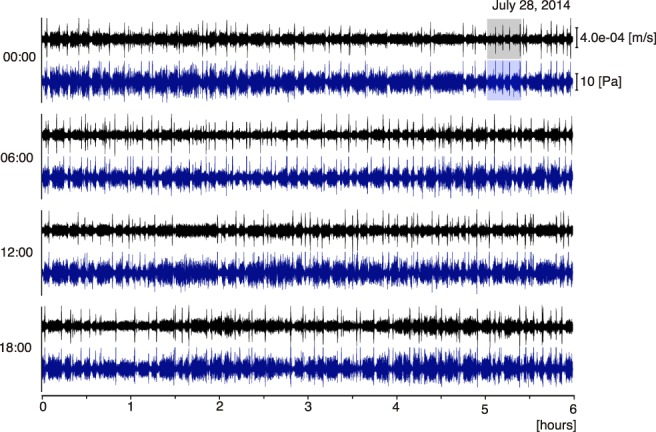
Vertical ground velocity seismogram at RFR (black) and infrasonic signal recorded by an element of the infrasound array (blue) during the 6 hours following the UTC time denoted on each y-axis on July 28, 2014. Both traces show a similar characteristic cycle in amplitude fluctuation. Grey and blue shaded periods are magnified in Figs 3b and 4a respectively.

**Figure 3:**
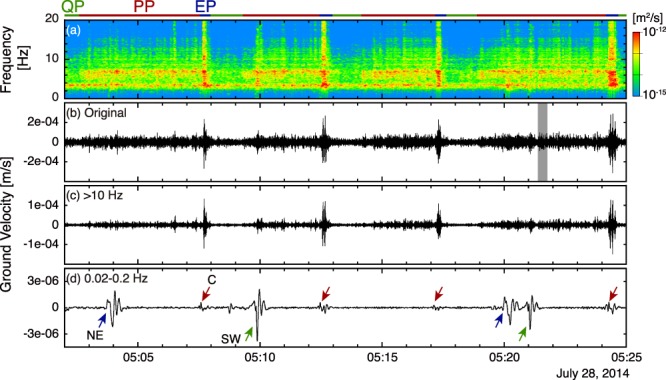
(**a**) Spectrogram of vertical ground velocity, (**b**) Seismogram of original vertical velocity, (**c**) High-pass filtered seismogram (>10 Hz), and (**d**) Band-pass filtered seismogram (0.02–0.2 Hz) of grey shaded interval in Fig. 2. The spectrogram was calculated using a Fast Fourier Transform of 512 data points (time window of 5.12 s with a sliding interval of 1 s) and smoothed by a Parzen window. The green, red, and blue horizontal bars at the top of the figure indicate the timing of QP, PP, and EP respectively. The red arrows indicate the timing of explosions at the C crater. Very-long-period (VLP) events associated with explosions occurred at different craters are indicated by blue (NE) and green (SW) arrows. Infrasound waveform in the same time window is shown in Fig. 4. The grey shaded period in Fig. 3b is magnified in Supplementary Fig. 1.

During our one-year long observation period, the waveforms recorded ten days before the lava outflow occurred on August 6 by the seismic and infrasonic sensors showed remarkably similarity (Fig. 2). Both signals clearly show similar changes in the amplitude which repeat in time following a cyclic pattern characterized by: (1) a quiet phase (QP) of low seismic and acoustic amplitudes (the green horizontal bars in Fig. 3), (2) a puffing phase (PP) (the red horizontal bars in Fig. 3), and (3) an explosion phase (EP) (the blue horizontal bars in Fig. 3). The high similarity of the amplitude modulation between the seismic and infrasonic records strongly suggests that the source responsible for this pattern is well coupled, both within the ground and the atmosphere and should be related to a source process well coupled with the atmosphere occurring at the surface of the magma column. We found that, particularly during the PP, analysis based on waveform correlation of the seismic signal revealed that the source location and mechanism were extremely stable (see Method for details).

**Figure 4:**
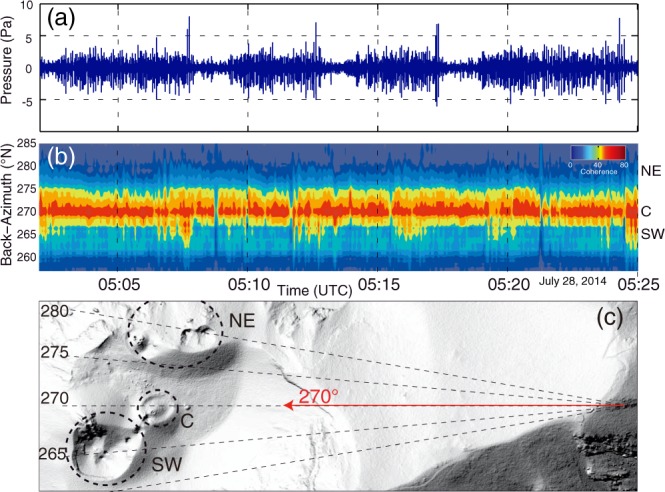
Location of acoustic activity revealed by the microphone array. (**a**) Infrasound waveform corresponding to the same time window with Fig. 3. (**b**) Temporal change in back azimuth of the infrasound signal. Colour scale represents signal coherence in array analysis. Coherent signal arrives from the direction of ~270°N throughout the QP-PP-EP cycle. (**c**) Projection of sound azimuth onto Digital Elevation Model. Red arrow indicates the corresponding infrasound azimuth which directs to the C crater. The map around the crater was created using Matlab ver. 7.5.0.338 (R2007b). Seismic waveform in the same time window is shown in Fig. 3.

**Figure 5:**
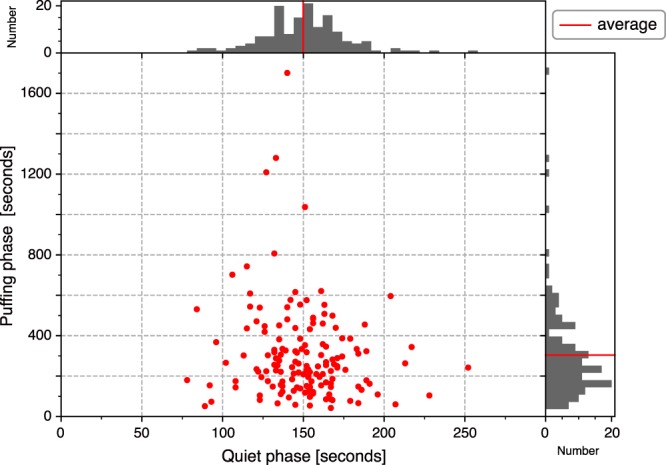
Relationship between the duration of the quiet phase (QP) and the puffing phase (PP) in each cycle. One day long data on July 28, 2014 shown in Fig. 2 were investigated and 150 cycles were identified. Duration of the QP is distributed in the narrow range of 149.85 ± 26.79 s and that of the PP widely varies in the range of 303.95 ± 233.05 s.

Interestingly, the duration of the QP was very stable, on average ~150 s (see Figs 2–5). The duration of the PP, on the other hand, varies within a few minutes and no correlation can be seen between the duration of the QP and the PP (Fig. 5). On July 28, 150 cycles were identified (see Figs 2 and 5).

The base frequency of seismic signal did not change during the cycle, remaining stable throughout the three different phases (QP, PP, and EP). Its range was limited in to between 2–4 Hz, which coincides with the spectral content of tremors at Stromboli^17,18^.

The PP was characterized by a series of repeating transient pulses (Fig. 3b,c), with high-frequency content in the 4–13 Hz range. These small pulses overlap with the low-frequency signal (2–4 Hz) in the spectrogram (Fig. 3a), suggesting that the high-frequency repeating pulses during the PP were superimposed onto the background volcanic tremor commonly recorded at Stromboli. Similar pattern of repeating pulses has been observed by a radiometer and infrasonic sensor only once before an occurrence of Strombolian explosion on June 19, 1999 (ref.^6^). These small pulses are related to the emission of small over-pressurized gas pockets at the surface of the magma column^19^, and represent the active degassing of the magma column at open-vent systems (puffing). This puffing represents the “active” expression of the continuous degassing of the magma column^8^.

At Stromboli, most puffing activity is located at the C crater, although it sometimes also occurs along the NE-SW elongated vents^10,16^. During the 2014 activity, infrasound signals came from the C crater (Fig. 4b,c). As the volcanic activity was quite high before the flank eruption^10^, signals from puffing were recorded not only by the infrasonic sensor, but also by the seismometer during the PP. Puffing activity at the C crater was very vigorous relative to usual periods, and could be classified as rapid spattering^20^. It should be noted here that small puffing activity never stopped and continued also during the QP. This is confirmed by the back-azimuth of infrasound signal which was pointing to the stable C crater direction of ~270°N (Fig. 4c). This fact indicates that the puffing continuously occurred with different amplitude (Fig. 4a) at the C crater throughout the cycles of QP-PP-EP.

The PP continued for variable periods on the order of several minutes, and then ended with a large transient EP associated with a Strombolian explosion at the C crater, and a very-long-period (VLP) event (red arrows in Fig. 3d). It means that Strombolian explosion coexisted with puffing at the C crater before the flank eruption. The small transient signals during the PP and large explosive event of the EP show similar waveforms, but the EP waveform displays slightly broader spectral peaks (EP in Fig. 3a). Seismic VLP events not related to this characteristic cycle showing different waveform and larger amplitude were also observed during large explosions occurring at other craters (VLPs with blue and green arrows in Fig. 3d). SO_2_ measurement of Strombolian explosion suggested that the signal amplitude of VLP scales with the volume of gas released during the explosion^21^ and this can explain the small VLP signal associated with the EP.

### Inflating magma column

**Figure 6:**
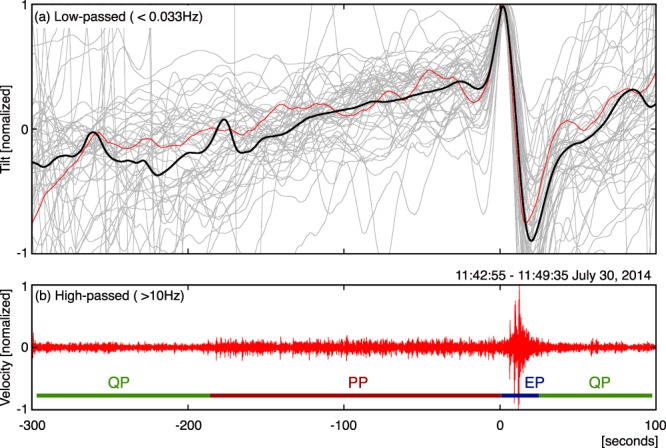
(**a**) Inflation-deflation cycle around the EP (*t* ~ 0) recorded by tiltmeter at CPL. The thick black line shows the stacked waveform of 50 normalized tilt traces (grey lines and red line). Each tilt trace was first filtered by a zero-phase low-pass filter with a corner frequency at 0.033 Hz and then normalized to the positive peak of the EP. Each trace was then aligned to the first positive peak and stacked to increase the signal-to-noise ratio and to eliminate the contribution ground deformation induced by explosions occurring at different craters. (**b**) One cycle of the signal (>10 Hz) recorded by a seismometer at RFR (11:42:55–11:49:35 on July30). Stacked tilt well correlates with the QP-PP-EP cycle of the seismic signal. Ground deformation during this signal is indicated by a red trace in (**a**).

Explosive activity at Stromboli has been associated with a cyclic ground deformation close to the summit area, indicating that explosions are often preceded by a slow inflation of the ground lasting ~200 s, followed by a sharp deflation during the explosive phase^22^. We analysed tilt data recorded at the CPL station during the cyclic seismic pattern. First, we processed the data with a low-pass filter (<0.033 Hz), before stacking it (Fig. 6a). Stacking shows that in our case, the EP is coincident with the sudden drop in the tilt, and that it is preceded by the gradual inflation of the ground lasting ~200 s. This inflation-deflation cycle is quite similar to that previously reported^22^, and seems to indicate that the PP is related to pressurization of the magma column probably induced by the increase of overpressurized gas bubbles rising in the conduit^23^ or by an increase in the magma-gas supply rate. The EP is then followed by a decrease of seismic amplitude, which corresponds to the 150 s QP.

## Discussion

Gas puffing represents persistent degassing activity of the magma column that is two times more efficient than a Strombolian explosion^24^, thus representing a major aspect of volcano dynamics. Explosions and puffing show significant short-term variability, indicating that the degassing activity of the magma column at Stromboli can be very sensitive to changes in the magma supply rate of the shallow feeding system^1,25^. Although small transient events due to puffing have previously been simultaneously recorded by infrasonic and radiometer sensors^6^, transient seismic signals excited by puffing have not been reported prior to this study. Generally, puffing would be expected to generate small pulses only in infrasonic records, but not in seismic records, which more commonly exhibit a continuous signal typically defined as a tremor^6,18,19^, because small seismic transients radiated by puffing repeating with short time interval (<1 s) are merged together due to the strong crustal heterogeneity of the elastic medium^26^.

We show that, in the period preceding the effusive eruption, these small transient pulses were also evident in the seismic records during the PP (see Fig. 3), and that infrasound pulses during the PP increased in amplitude relative to those in the QP (see Fig. 4). The puffing activity preceded the Strombolian explosion and was associated with an increase in ground deformation (Fig. 6), suggesting a progressive pressurization of the magma column in response to increase in gas flux before the explosion. After the occurrence of the explosion, the seismic pattern repeated in cycles of variable durations (1 to 10 minutes), but every cycle showed the almost same 150 s-long QP, during which only small puffing occurs (see Figs 2, 4 and 5).

These seismicity cycles are in agreement with gas flux measurements recorded using UV camera imaging, indicating that each explosion was associated with a significant SO_2_ flux cycle, lasting 300–700 s (ref.^24^). This caused a quasi-periodic SO_2_ degassing behaviour with a duration consistent with the seismicity cycle.

The physical mechanisms of puffing and explosions have been intensively studied by observational, theoretical, and experimental approaches^3–6,8,19^. The dynamics of volcanic gas ascending in the conduit plays a central role in the degassing styles of both puffing and explosions. Our common understanding of these two different degassing styles is that puffing and explosion are related to different gas flux regimes. In two-phase flow in vertical pipes, the flow regimes are often classified as bubble, slug, churn, and annular flow^27^. Whereas puffing is most probably associated to bubble flow, explosions likely reflect a gas slug flow regime in the conduit^6,19,28,29^. The bubble-to-slug transition in the flow regime is commonly linked to the growth of void waves, and typically occurs at a void fraction of ~0.25 (ref.^30^). Experimental evidence indicates that the transition between these two regimes occurs when the gas superficial velocity (i.e. gas flux) increases^31^.

The end of explosive activity at the crater on August 6 (ref.^10^) lead to a sudden stop of the cyclic seismicity. On the next day, a new lateral vent opened in SDF, and volcanic activity changed into the effusive discharge of large amount of magma. After this drastic change in volcanic activity, the cyclic seismicity pattern could no longer be detected.

We observed cyclic signals from July 27 to August 6, 2014 at Stromboli, immediately prior to the effusive eruption on August 7, 2014; explosive activity at the summit crater was at a high level. The signals we recorded reflect the cyclic behaviour of rising volcanic gas in the conduit below the central (C) crater, consisting of three phases: (1) a quiet phase (QP), with very small pulses only in infrasound, (2) a vigorous puffing phase (PP), featuring repeating high-frequency pulses both in seismogram and infrasound, and (3) an explosion phase (EP). This characteristic cycle in the seismic signal was observed only in the period preceding the effusive eruption. The signal disappeared after the transition from the summit activity to the flank eruption. Our observations further demonstrate that explosive activity at Stromboli is modulated by cyclic volcanic degassing behaviour, over timescales of several minutes. We conclude that these seismicity cycles represent cyclic changes of the gas flux regime in the shallow magma column, associated with increases in the magma-gas supply input rate before the effusive eruption. The cyclic behaviour of explosive degassing is an important factor in the degassing dynamics of open-vent volcanoes.

## Methods

### Detection of the pulse in the PP

We here demonstrate that the PP in seismogram is composed by numerous repeating pulses that have almost identical amplitudes (Supplementary Fig. 1), and which are most probably excited by vigorous puffing at the C crater. Our detailed analysis of the cycle indicates that the PP is characterized by a series of individual pulses with very similar waveforms to each other. Once we isolated a master pulse, we were able to isolate each repeating pulse using a waveform cross-correlation. Here we introduce the idea of the Network Correlation Coefficient (NCC)^32^, and define the modified NCC time function at one station as:

1 $$NCC(t)\equiv \frac{1}{N}\sum _{i=1}^{N}\,{C}_{i}(t)$$

where *C*_*i*_(*t*) is the time history of the correlation coefficient of the *i*-th component, and *N* is the total number of components at the station. In this analysis, we used three component seismograms recorded at RFR and PZZ (*N* = 3) and two component tilt datasets at CPL (*N* = 2). The data length of a master event was ~2 s (201 samples), which is long enough to contain one pulse. Before the NCC function was calculated, both the master waveform and dataset were high-pass filtered using a two-pole zero-phase Butterworth filter with a corner frequency of 10 Hz to emphasize the characteristic high-frequency content of the pulses. After several trials, the threshold value for event detection at RFR and PZZ was defined as 0.6, whereas the threshold at CPL was 0.5 because the tiltmeter at CPL is not as sensitive to high-frequency signals as the seismometer. To avoid duplicate detections, when the NCC was detecting a pulse with a NCC larger than the threshold, all other pulses occurring one second before and after this pulse were excluded from the event search (Supplementary Fig. 2).

We used a pulse that occurred in the PP at 21:21:56 on July 31 as the template event (*t* ≈ 136 in Supplementary Fig. 2); the NCC value for this template pulse certainly becomes one from its definition in Eq. (1) (orange arrows in Supplementary Fig. 2). Our method was able to identify most of the pulses recorded at RFR and PZZ, whereas at CPL the number of pulses identified was much smaller because of the low sensitivity of the tiltmeter to the high-frequency ground motion. Using a different pulse as the template event delivered similar results. At the RFR station, pulses were also detected during the EP (*t* ≈ 190 in Supplementary Fig. 2) indicating the stability of the waveform among different phases. However, since the pulses during the EP had a broad spectral content (EP in Fig. 3a), the detection rate became lower than during the PP.

We analysed the seismic data recorded from May 19 to the end of August. As shown in Supplementary Fig. 3, a high NCC value was obtained from the middle of June, although the characteristic cyclic pattern was only identified after July 26. Since the pulse was excited by puffing activity, which is persistent at the C crater, it is quite natural that the similar pulses were detected before the cyclic pattern begins. The total number of pulses detected in the PP remained similar even when the master pulse used to calculate the NCC was changed (198736, 230016, and 172684; Supplementary Fig. 3). Furthermore, the NCC amplitude does not appear to be dependent on the choice of the master pulse, showing similar values and long-term fluctuations for different master pulses (Supplementary Fig. 3). The NCC reached values of ~0.8 from the middle of June to the middle of July almost following the increase of seismic tremor amplitude (Supplementary Fig. 3a) which indicates that the pulses in seismic record have a sufficiently large signal to noise (S/N) ratio to be detected in this period.

The pulses and the cycles disappear after 12:00 on August 6 (Supplementary Fig. 3d,e), coinciding with the onset of the lateral intrusion of lava preceding the effusive eruption in the Sciara del Fuoco, and with the end of explosive activity at the crater^10,11^.

### Locating the source

In terms of source location, due to the small amplitude of the pulses (i.e., small S/N ratios), it was quite difficult to determine the absolute position of the seismicity (see Supplementary Fig. 1). However, relative changes of the source can be evaluated by examining time differences in the NCC lag among the three stations (RFR, PZZ, and CPL). Supplementary Fig. 4a shows 10 second-long NCC functions at the three stations using a pulse that occurred (in the PP) at 21:25:20 on July 27 (the template 2) as the template. The template pulse is included in this 10 s time window (indicated by the orange arrow). Three peaks can be recognized about 4 seconds after template 2 arrives, almost at the same time (indicated by the black arrow). Supplementary Fig. 4b,c show the same plots as Supplementary Fig. 4a, but with different time windows. We used the same template pulse (the template 2) for these calculations. Supplementary Fig. 5a shows the histograms of the time differences in the NCC peaks on July 27. Supplementary Fig. 5b,c show the same histogram as Supplementary Fig. 5a but with a different date.

The time difference at the position of the maximum NCC value was always within the range of one sample (0.01 second) at all three stations. When we assume shear wave velocity *V*_*s*_ = 0.27–1.14 km/s (ref.^33^) and the elevation of source *H*_*c*_ ≈ 770 m (the elevation of crater)^10^, the sources of the pulses in the PP were concentrated within 30 m. That is, the hypocentre of the pulses did not change significantly.

The similarity of the pulse waveform, together with the shallow location of the source, indicates that the source location and dynamics did not change in time throughout the observation period. This is consistent with the other previous researches, which define seismic tremors at Stromboli as being linked to the active degassing (puffing) of the magmatic column^6,18,19^. Infrasound array processing shows, in fact, that acoustic pressure related to puffing activity followed the same NCC fluctuations, and that source of the infrasound was stable and was located within the C crater (Fig. 4).

## Supplementary information

Supplementary information for Method

## Data Availability

The seismic and tilt data analysed during the current study are not publicly available due to a data volume but are available from the corresponding author on reasonable request.

## References

[1] Ripepe, M., Delle Donne, D., Harris, A. J., Marchetti, E. & Ulivieri, G. Dynamics of Strombolian Activity. In *The Stromboli volcano: An integrated study of the 2002–2003 eruption* (eds Calvari, S., Inguaggiato, S., Puglisi, G., Ripepe, M. & Rosi, M.) 39–49, 10.1029/182GM05 (American Geophysical Union, 2008).

[2] Chouet B (2003). Source mechanisms of explosions at Stromboli Volcano, Italy, determined from moment-tensor inversions of very-long-period data. J. Geophys. Res..

[3] Gaudin D (2017). Integrating puffing and explosions in a general scheme for Strombolian-style activity. J. Geophys. Res..

[4] Jaupart C, Vergniolle S (1988). Laboratory models of Hawaiian and Strombolian eruptions. Nature.

[5] Ripepe M, Ciliberto S, Della Schiava M (2001). Time constraints for modeling source dynamics of volcanic explosions at Stromboli. J. Geophys. Res..

[6] Ripepe M, Harris AJL, Carniel R (2002). Thermal, seismic and infrasonic evidences of variable degassing rates at Stromboli volcano. J. Volcanol. Geotherm. Res..

[7] Barberi F, Rosi M, Sodi A (1993). Volcanic hazard assessment at Stromboli based on review of historical data. Acta Vulcanol..

[8] Harris A, Ripepe M (2007). Temperature and dynamics of degassing at Stromboli. J. Geophys. Res..

[9] Ripepe M (2017). Forecasting effusive dynamics and decompression rates by magmastatic model at open-vent volcanoes. Scientific Rep..

[10] Valade S (2016). Tracking dynamics of magma migration in open-conduit systems. Bull. Volcanol..

[11] Rizzo AL (2015). The 2014 effusive eruption at Stromboli volcano (Italy): Inferences from soil CO_2_ flux and ^3^He/^4^He ratio in thermal waters. Geophys. Res. Lett..

[12] Zakšek K, Hort M, Lorenz E (2015). Satellite and ground based thermal observation of the 2014 effusive eruption at Stromboli Volcano. Remote Sens..

[13] De Martino S, Falanga M, Palo M, Montalto P, Patanè D (2011). Statistical analysis of the volcano seismicity during the 2007 crisis of Stromboli, Italy. J. Geophys. Res..

[14] Ripepe M (2015). Volcano seismicity and ground deformation unveil the gravity-driven magma discharge dynamics of a volcanic eruption. Nature Comm..

[15] Landi P, Marchetti E, La Felice S, Ripepe M, Rosi M (2011). Integrated petrochemical and geophysical data reveals thermal distribution of the feeding conduits at Stromboli volcano, Italy. Geophys. Res. Lett..

[16] Ripepe M, Marchetti E, Ulivieri G (2007). Infrasonic monitoring at Stromboli volcano during the 2003 effusive eruption: Insights on the explosive and degassing process of an open conduit system. J. Geophys. Res..

[17] Chouet B (1997). Source and path effects in the wave fields of tremor and explosions at Stromboli Volcano, Italy. J. Geophys. Res..

[18] Ripepe M, Poggi P, Braun T, Gordeev E (1996). Infrasonic waves and volcanic tremor at Stromboli. Geophys. Res. Lett..

[19] Ripepe M, Gordeev E (1999). Gas bubble dynamics model for shallow volcanic tremor at Stromboli. J. Geophys. Res..

[20] Houghton B (2016). Stronger or longer: Discriminating between Hawaiian and Strombolian eruption styles. Geology.

[21] McGonigle AJS, Aiuppa A, Ripepe M, Kantzas EP, Tamburello G (2009). Spectroscopic capture of 1 Hz volcanic SO_2_ fluxes and integration with volcano geophysical data. Geophys. Res. Lett..

[22] Genco R, Ripepe M (2010). Inflation-deflation cycles revealed by tilt and seismic records at Stromboli volcano. Geophys. Res. Lett..

[23] James MR, Lane SJ, Chouet B, Gilbert JS (2004). Pressure changes associated with the ascent and bursting of gas slugs in liquid-filled vertical and inclined conduits. J. Volcanol. Geotherm. Res..

[24] Tamburello G, Aiuppa A, Kantzas EP, McGonigle AJS, Ripepe M (2012). Passive vs. active degassing modes at an open-vent volcano (Stromboli, Italy). Earth Planet. Sci. Lett..

[25] Marchetti, E. *et al*. Gas flux rate and migration of the magma column in *The Stromboli volcano: An integrated study of the 2002–2003 eruption* (eds Calvari, S., Inguaggiato, S., Puglisi, G., Ripepe, M. & Rosi, M.) 259–267, 10.1029/182GM21 (American Geophysical Union, 2008).

[26] Kedar S, Sturtevant B, Kanamori H (1996). The origin of harmonic tremor at Old Faithful geyser. Nature.

[27] Hewitt, G. F. Flow regimes in *Handbook of Multiphase Flow System* (ed. Hetsroni, G.) 2-1–2-136 (Hemisphere Publishing Corp., 1982).

[28] Vergniolle S, Brandeis G (1994). Origin of the sound generated by Strombolian explosions. Geophys. Res. Lett..

[29] Manga M (1996). Waves of bubbles in basaltic magmas and lavas. J. Geophys. Res..

[30] Song CH, No HC, Chung MK (1995). Investigation of bubble flow developments and its transition based on the instability of void fraction waves. Int. J. Multiph. Flow.

[31] Taitel Y, Bornea D, Dukler AE (1980). Modelling flow pattern transition for steady upward gas-liquid flow in vertical tubes. AIChE J..

[32] Gibbons SJ, Ringdal F (2006). The detection of low magnitude seismic events using array-based waveform correlation. Geophys. J. Int..

[33] Chouet B (1998). Shallow velocity structure of Stromboli Volcano, Italy, derived from small-aperture array measurements of strombolian tremor. Bull. Seismol. Soc. Am..

[34] Wessel, P. & Smith, W. H. F. New, improved version of the Generic Mapping Tools released. *Eos Trans. AGU* **79**, 579 (1998).

