# Supplementary material for: Gas flux cyclic regime at an open vent magmatic column inferred from seismic and acoustic records
Source: Sci Rep. 2019 Apr 5;9:5678. doi: 10.1038/s41598-019-42033-z (PMC6450954; doi:10.1038/s41598-019-42033-z)
Supplement: Supplementary file 1 — Supplementary information for Method [file 41598_2019_42033_MOESM1_ESM.pdf]

Supplementary of
Gas flux cyclic regime at an open vent magmatic column inferred from seismic and acoustic records

Gen Kondo[1], Hiroshi Aoyama[2,*], Takeshi Nishimura[3], Maurizio Ripepe[4], Giorgio Lacanna[4], Riccardo Genco[4], Ryohei Kawaguchi[5], Taishi Yamada[6], Takahiro Miwa[6], Eisuke Fujita[6]

[1]Graduate School of Science, Hokkaido University, Sapporo, 060-0810, Japan

[2]Faculty of Science, Hokkaido University, Sapporo, 060-0810, Japan

[3]Graduate School of Science, Tohoku University, Sendai, 980-8578, Japan

[4]Dipartimento di Scienze della Terra, Università di Firenze, Firenze, 50121, Italy

[5]Meteorological Research Institute, Japan Meteorological Agency, Tsukuba, 305-0052, Japan

[6]National Research Institute for Earth Science and Disaster Resilience, Tsukuba, 305-0006, Japan

*Corresponding author: Hiroshi Aoyama (aoyama@sci.hokudai.ac.jp)

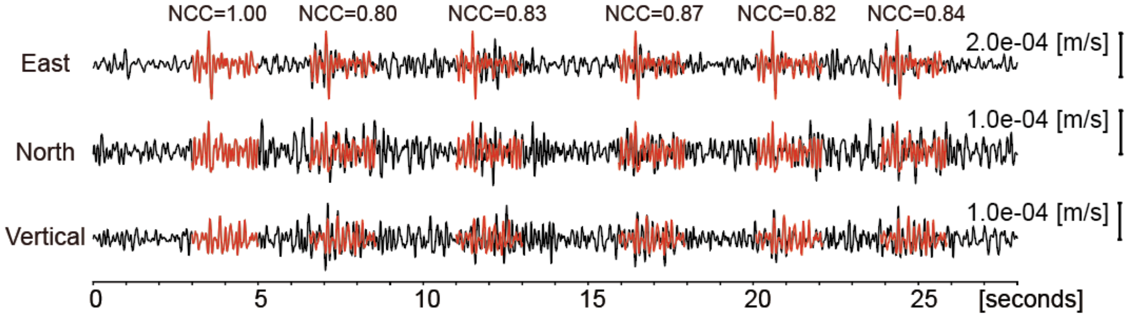

Figure S1. Seismic records during the PP. Three-component velocity waveforms during the grey shading period in Figure 3b. One master pulse ($NCC = 1$) in the PP is superimposed on the other pulses that were automatically detected in this study. The NCC is an index indicating the degree of waveform similarity.

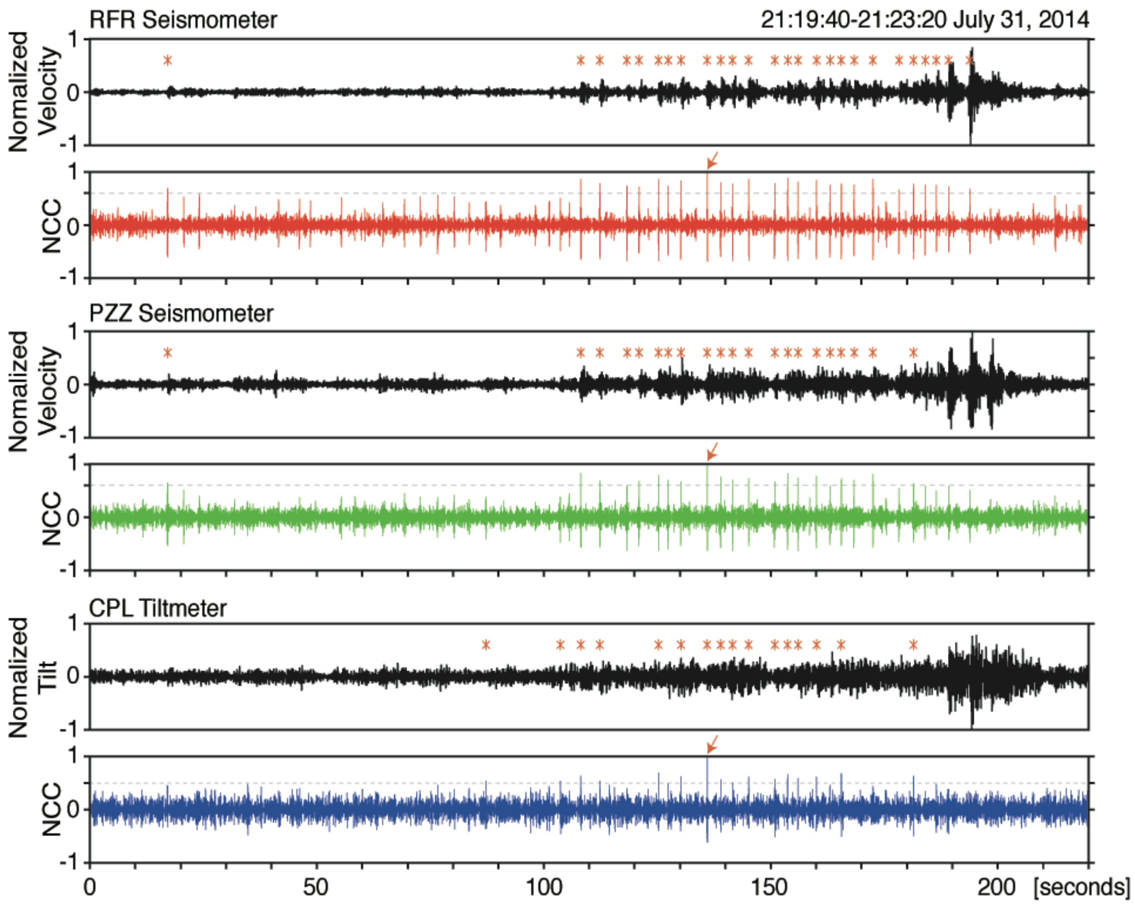

Figure S2. An example of pulse detection over one cycle. The black traces show seismic waveform (RFR and PZZ) and tilt records (CPL). The component was arbitrarily selected. These traces were filtered by the same zero-phase high-pass filter with corner-frequency at 10 Hz. The coloured traces express the NCC function at each station. Orange arrows indicate a template event used in this example at 21:21:56. Orange stars express the detected pulses with NCC values exceeding the given threshold (dashed grey line). The chosen threshold values were 0.6 for RFR and PZZ and 0.5 for CPL, after a process of trial and error.

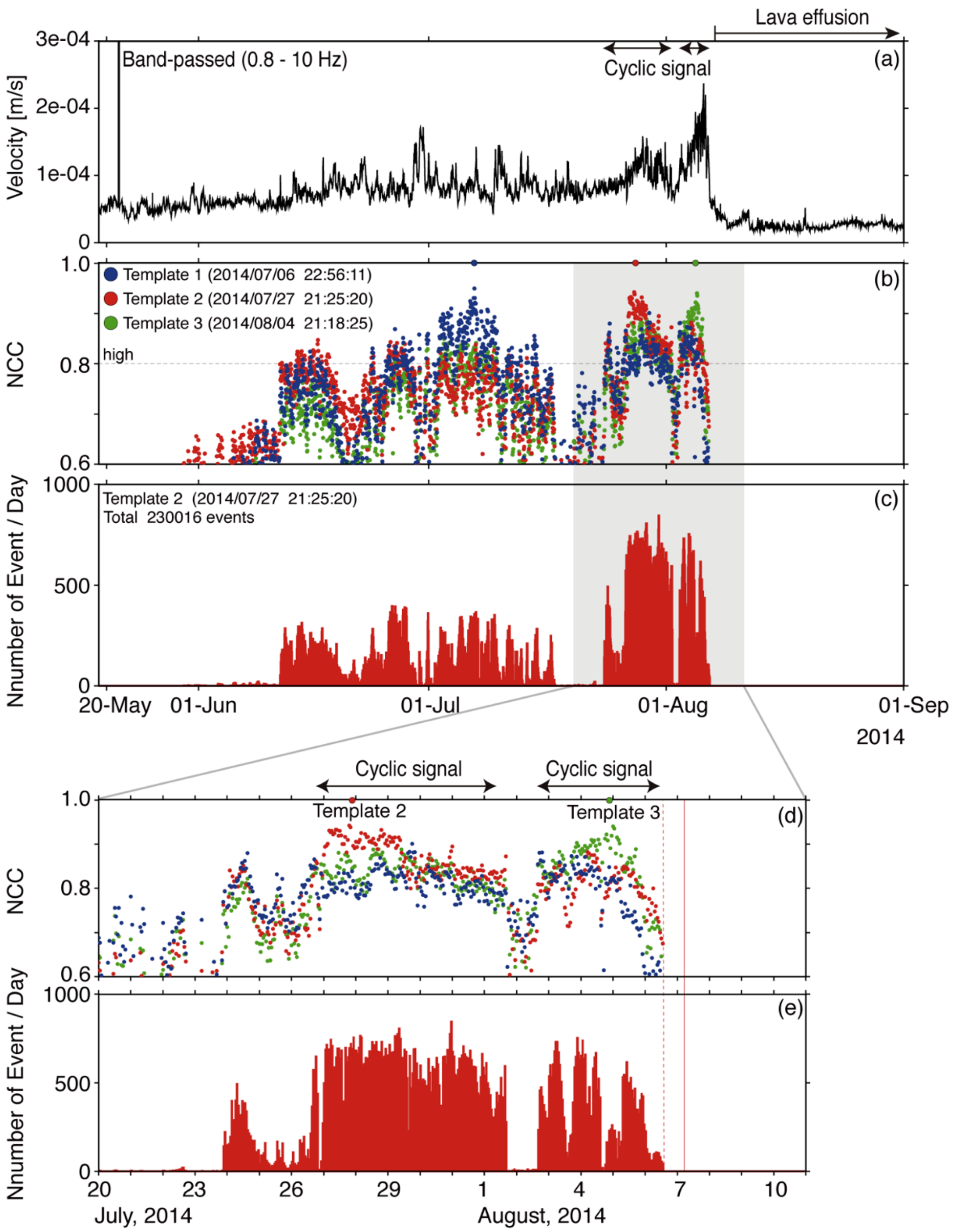

Figure S3. Long-term activity of puffing. (a) Seismic tremor amplitude (0.8 – 10 Hz) at RFR. (b) Long-term change in NCC values of RFR for three different templates, from the beginning of our observation in late May 2014 to the end of August. To maintain the clarity of the plot, the maximum NCC value for every hour was extracted from the event

lists and plotted here. (c) Number of detected pulses per hour in the PP from May 19 to August 31, 2014. We used the event list at RFR generated by the template pulse (in the PP) at 21:25:20 on July 27 (the template 2). (d)(e) Magnified plot around the lava effusion event (shaded period in (b) and (c)). The dashed red bar indicates the onset of lava overflow from the crater terrace (at 12:00 on August 6, 2014). The solid red bar indicates when the new effusive vent opened (at 05:15 on August 7, 2014). The double-headed arrow indicates the period for which the cyclic seismicity pattern was observed.

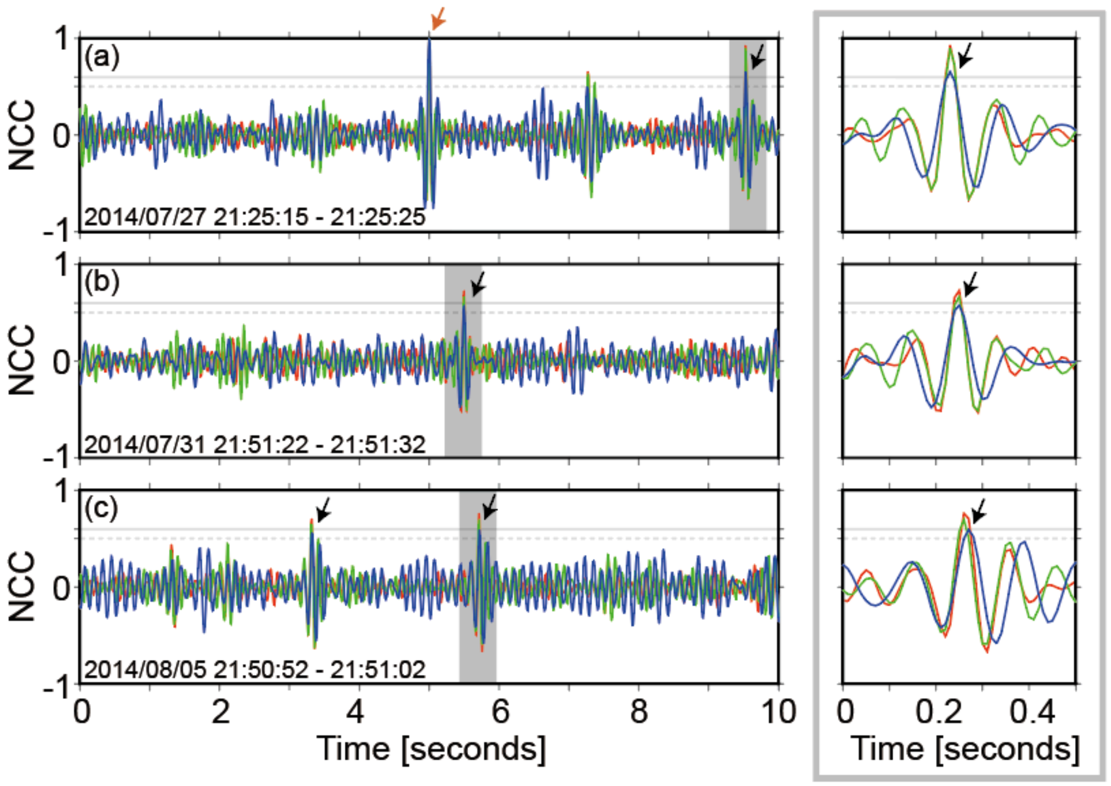

Figure S4. Examples of NCC functions. We used a pulse (in the PP) at 21:25:20 on July 27 (the template 2) as the template. Here we show 10 s-long (left) and 0.5 s-long (right) NCC functions on July 27 (a), on July 31 (b), and on August 5 (c) as examples. Red, green and blue lines correspond to RFR, PZZ and CPL station respectively. The functions on the right-hand side of the figure are enlarged functions of grey shaded periods in the left. The solid grey line indicates the threshold value at RFR and PZZ ($NCC = 0.6$) and the dashed gray line indicates the threshold value at CPL ($NCC = 0.5$). The orange arrow indicates the template event and the black arrows indicate the detected events.

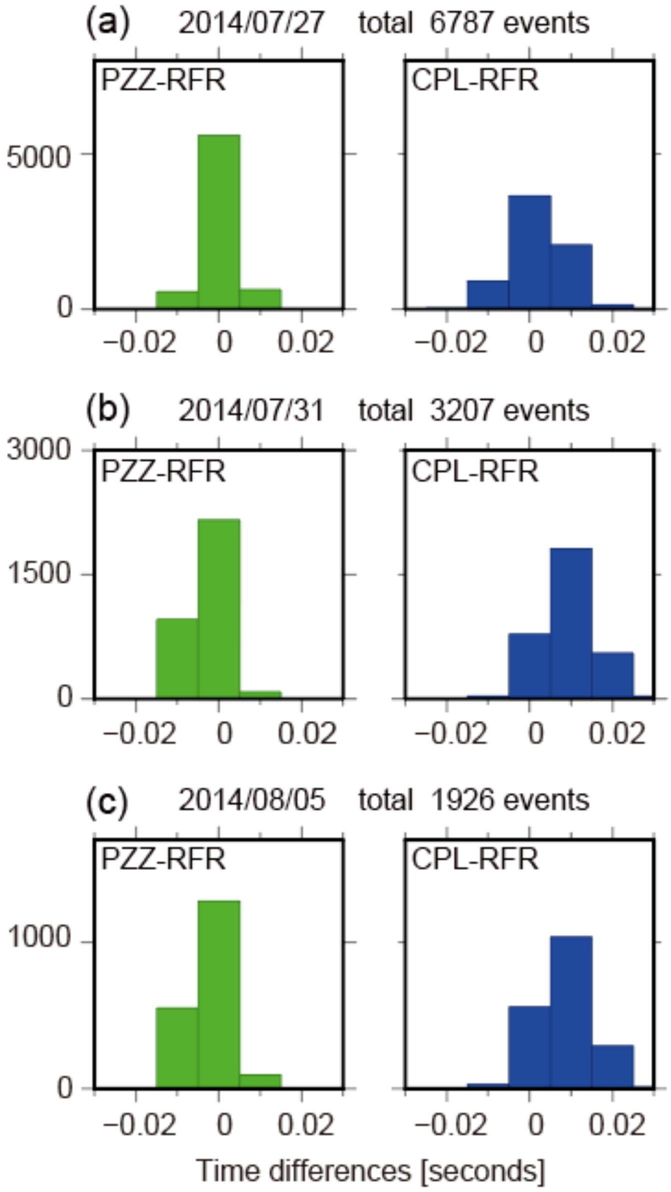

Figure S5. Histograms of the time differences in the NCC peaks. We used a pulse (in the PP) at 21:25:20 on July 27 (the template 2) as the template. Here we show histogram of one-day on July 27 (a), on July 31 (b), and on August 5 (c). The green and blue histograms show the time differences between the peak NCC value at RFR and the peak NCC values at PZZ and CPL respectively.
